# Impact of Resected Gastric Volume on Postoperative Weight Loss after Laparoscopic Sleeve Gastrectomy

**DOI:** 10.1155/2019/3742075

**Published:** 2019-12-01

**Authors:** Stefano D'Ugo, Vittoria Bellato, Emanuela Bianciardi, Paolo Gentileschi

**Affiliations:** ^1^Department of General Surgery, Vito Fazzi Hospital, Lecce, Italy; ^2^Department of Minimally -Invasive Surgery, University of Rome Tor Vergata, Italy; ^3^Department of Systems Medicine, University of Rome Tor Vergata, Italy; ^4^Department of Surgery, Obesity Unit. University of Rome Tor Vergata, Italy

## Abstract

Among the bariatric surgery community, it has recently emerged the idea of a possible association between resected gastric volume (RGV) and weight loss after laparoscopic sleeve gastrectomy (LSG). If the size of the sleeve depends on the bougie caliber, the resected volume of the stomach remains something which is not possible to standardize. The aim of the study was to investigate a possible relationship between RGV and weight loss after LSG. We developed a mathematical method to calculate the RGV, based on the specimen size removed during LSG. Ninety-one patients (63 females and 28 males) affected by morbid obesity were included in the study. They underwent LSG between 2014 and 2016. Mean preoperative BMI was 45 ± 6.4. At 1 year after LSG, the mean BMI was 30 ± 5.3 and the EWL% was 65 ± 20.2. The statistical analysis of RGV, BMI, and EWL% at 1-year follow-up did not find any correlation between the volume of stomach removed and the weight loss after LSG. Further studies in the future should clarify the potential role of RGV during LSG. This trial is registered with ClinicalTrials.gov NCT03938025.

## 1. Introduction

Morbid obesity is one of the most concerning public health issues in the world. Excess body weight is the sixth most important risk factor contributing to the overall burden of diseases worldwide. 1.1 billion adults and 10% of children are now classified as overweight or obese [1].

The main goal of the surgical therapy is to achieve long-standing results in terms of weight loss, amelioration of comorbidities, and avoidance of weight regain.

Laparoscopic sleeve gastrectomy (LSG) as a standalone procedure [2] has been associated with significant improvement of health status regarding diabetes, hypertension, metabolic syndrome, and related diseases. Literature data showed a mortality comparable to that of laparoscopic cholecystectomy and a reported mean percentage excess body weight loss (EBWL) of 59.3% after 1 year [3].

Recently, the idea of an association between the resected gastric volume (RGV) and the weight loss after LSG [4–9] emerged, although each author provided different methods and approaches to assess the factors' relationship. This led to difficulties in the comparison of the results.

Two main issues have emerged in those studies: the first one is linked to the standardization of the procedure, including the bougie caliber to shape the gastric sleeve [10–12]; the second one is related to the possible methods used to assess the RGV.

Moreover, other authors suggested to record the results after longer-term follow-up, in order to consider also the possible effect of the sleeve dilatation on weight loss or weight regain [13].

Given that the size of the sleeve should be based on the caliber of the bougie, the RGV represents the nonstandardized variable.

In this study, we investigate a possible relationship between RGV and weight loss after LSG, using a new a mathematical method we developed to calculate the RGV, and we compare our results with previously published data.

## 2. Materials and Methods

This study was a retrospective analysis of prospectively collected data of morbidly obese patients listed for bariatric surgery in our unit. We included patients undergoing LSG as standalone procedure over a period between 2014 and 2016. Patients were studied preoperatively with a multidisciplinary workup including specialist counselling (surgery, endocrinology, internal medicine, psychiatry, anesthesiology), gastrointestinal endoscopy, and performance status evaluation. Patients were well informed about the surgical procedure, with all potential advantages and possible complications and side effects.

Indications for LSG were established according to the international guidelines: BMI over 40 kg/m^2^ with no diabetes, BMI between 35 and 40 kg/m^2^ with associated comorbidities, patients with greater BMI and high surgical risks or refusing a more complex procedure like Roux-en-Y gastric bypass, patients with previous bowel surgery, and young patients refusing gastric banding. Diabetic patients were first selected for Roux-en-Y gastric bypass and then to LSG if they refused the gastric bypass. Specific contraindications, apart from the general contraindications to bariatric surgery, were severe and documented gastroesophageal reflux disease and previous gastric surgery.

### 2.1. Surgical Technique

All LSGs were performed by the same surgical team using a standard technique.

Abdominal insufflation was set at 15 mmHg. Using a dissecting coagulator, the greater curvature of the stomach was mobilized at a point 3 cm proximal to the pylorus. The lesser sac was entered, staying close to the wall of the stomach; the greater curvature ligaments were divided all the way up to the angle of His. The left crus of the diaphragm was exposed to delineate the gastroesophageal junction and to facilitate complete resection of the gastric fundus. Retrogastric adhesions were taken down to allow for complete mobilization of the stomach, to eliminate any redundant posterior wall of the sleeve, mainly at the fundus. A 36-40 Fr was inserted and positioned close to the lesser curvature, as a calibration for the gastric resection.

Gastric transection by a mechanical stapler began at a point 5 cm proximal to the pylorus, leaving the antrum and preserving the gastric emptying. Care was taken not to narrow the stomach at the angulus. The entire staple line was inspected for bleeding and tested for leak insufflating the gastric tube with methylene blue and saline.

On the first postoperative day, if the patient was comfortable and observations were stable, a liquid diet was started. In absence of signs of complication, patients were discharged as soon as they could walk and drink, usually on the 2nd postoperative day. Patients were given a liquid diet for two weeks and followed-up in the outpatient clinic for years by the multidisciplinary team.

The success of surgery was assessed at 1, 3, 6, and 12 months postoperatively, recording the weight, BMI, and EWL%. The latter as a ratio between the initial weight and weight at follow-up, and the initial weight and ideal weight (Lorenz formula).

In order to evaluate the longer-term effect of LSG and to assess a possible relationship between RGV and weight loss, we considered a follow-up of at least 1 year.

### 2.2. Resected Gastric Volume

**Figure 1:**
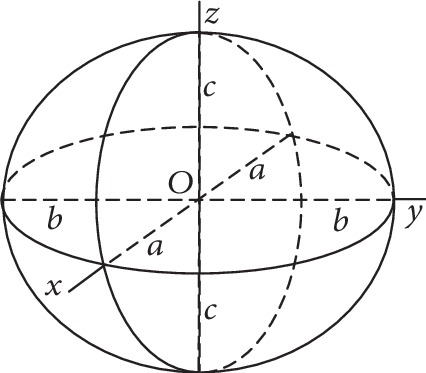
Geometrical characteristics of the ellipsoid.

Our study is aimed at finding a 3D geometrical figure which would adapt to the conformation of the resected stomach. If we consider the surgical section devoid of surgical suture margins, opened and laid out, the closest resembling figure is a hemiellipsoid (Figure 1).

The ellipsoid represents each second-order surface given by the mathematical equation:

(1) $$\begin{array}{c} \frac{x^{2}}{{a2}_{1}}+\frac{y^{2}}{a_{2}2}+\frac{z^{2}}{a_{3}2}-1=0 \end{array}$$

The volume of the ellipsoid can be calculated by applying the following formula, gathering the measurements of the resected gastric section from the pathologist:

(2) $$\begin{array}{c} V=\frac{4}{3}\pi abc⟶\frac{V}{2}=\frac{2}{3}\pi abc \end{array}$$

Given an approximation of the width to the fourth part of the circumference, which formula is represented as circumference = 2*πr*, we can get the *c* semiaxis as the radius *r* of the circumference, and it will correspond also to the *a* semiaxis as in the following equation:

(3) $$\begin{array}{c} \frac{\text{Circumference}}{4}=\text{width}=\frac{2\pi r}{4}=\frac{\pi r}{2}⟶r=c=a=\frac{2x\text{width}}{\pi }. \end{array}$$

With this mathematical model, it is possible to obtain the volume of the resected stomach during LSG.

A Microsoft Excel database was created using the Statistical Package for Social Science (SPSS ver. 15.0). The descriptive sample analysis evaluates mean values ± standard deviation with Gaussian distribution; the confirmation was given by Kolgomorov-Smirnov tests or histograms.

The comparison of continuous variables was performed using ANOVA (Analysis of Variance). The analysis of correlation between two variables (resected volume and weight, BMI, and EWL% at 1 year) was performed using the Pearson Correlation Coefficient (*R*). A *p* value < 0.05 was considered statistically relevant.

## 3. Results

Ninety-one patients (63 females and 28 males) satisfied the inclusion criteria. After revision of the analytical data, seventy patients with complete follow-up were included in the statistical analysis (44 females and 26 males). The mean age was 43.8 ± 8.3 years. The mean global preoperative weight was 132.7 ± 26.9 Kg, and BMI was 45.39 ± 6.4 Kg/m^2^. The male group showed greater numbers both in terms of weight (155 ± 26 Kg) and BMI (47.2 ± 7.9 Kg/m^2^).  Table 1 summarizes all the preoperative data.

After surgery, at 1-year follow-up, we observed a significant reduction of all the indexed parameters. The mean BMI was 30.1 ± 5.3 Kg/m^2^, with a mean global weight of 87.1 ± 20 Kg. The global mean EWL% was 65.1, with higher values in male patients.  Table 2 reports postoperative data 1 year after LSG.

The statistical evaluation of a possible relationship between RGV and weight loss showed no correlation between the two factors. In particular after 1 year, this was valid both for the EWL% (*p* value = 0.168, *R* = 0.167) and for the BMI (*p* value = 0.156, *R* = −0.172).  Table 3 shows the detailed results.

**Figure 2:**
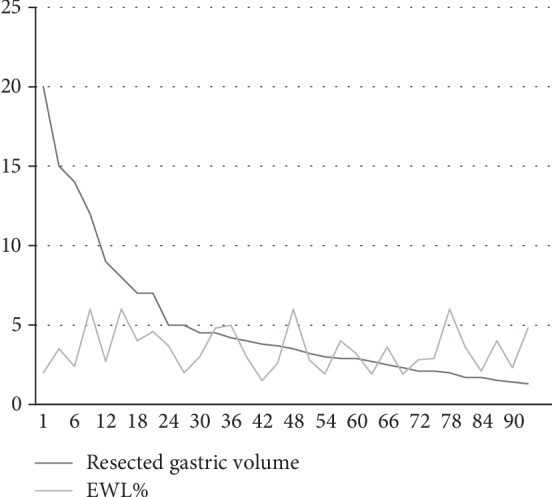
EWL% vs resected gastric volume trend at 1 year.

Figure 2 highlights on a diagram how, after plotting the trend of EWL% and RGV 12 months after LSG, there is a casual distribution of data.

## 4. Discussion

Evaluating how much the gastric reservoir size affects the weight loss in patients undergoing LSG is still an open question, since no ideal resection volume has been established yet.

Given the increasing popularity of LSG as a primitive surgical intervention, the clarification of a relationship between resected gastric volume and weight loss could have a major impact on the surgical outcomes.

Regarding this topic, at least two major factors should be considered. First of all, there is a lack of long-term follow-up data, with less than 10% of the literature describing results beyond 36 months. Second, there is no standardized method for the evaluation of both the volume of the sleeve and the volume of the stomach removed.

The use of criteria related to the bougie size is not really appropriate, because the orogastric tube is not the only factor impacting the final sleeve volume. We should also consider data like how close the resection is performed and how many centimeters from the pylorus. Also, the reinforcement of the staple line could further reduce the size of the sleeve.

In terms of distance from the pylorus, we have to consider that, in order to maintain an appropriate gastric emptying, many surgeons perform resections 4-6 cm from this valve, while others go closer to obtain a smaller gastric tube.

Some authors calculated the volume of the sleeve during the postoperative period with indirect radiological measures. The CT scan in the evaluation of the sleeve volume is a relatively precise method, as it allows 3D reconstructions of the gastric tube [14]. However, it is associated to obvious disadvantages, such as elevated costs and possible side effects (i.e., vomit or contrast allergy). Moreover, patients have a reduced compliance in the immediate postoperative period, with the risk to deliver an insufficient volume of oral contrast, resulting in inadequate sleeve distension and measurement [15].

More recent investigations moved the attention towards the volume of the stomach resected during LSG, in order to identify if this can impact the surgical results.

Different methods have been used to assess the RGV, despite none of them has been recognized as accurate and has been validated. Insufflation of the specimen with water under pressure followed by manometric tests is included; others recorded the volume of saline required to obtain a complete distention of the specimen. In all the cases, however, the evaluation is not realistic, being influenced by many factors like for example the elastic resistance of the gastric wall and the impact of the staple line on the resistance itself.

In our study, we developed a mathematical method which is simple and fast to apply to evaluate the RGV after LSG. The measurement is based on the pathology results and then does not require any nonvalidated technique to obtain a surrogate of the volume. The formula considers the RGV as a 3D geometrical figure, so it means that the method is easily reproducible in all the patients.

Up to date, in the scant literature available on this topic, there is no agreement on the possible relationship between RGV and weight loss. There are studies reporting a higher weight loss in case of larger RGV, but on the opposite side, others did not identify a possible role of this volume.

Obeidat et al. reported the results of 73 patients at 1-year follow-up after LSG, recording the volume of the resected stomach and the EWL%. This study showed a significant correlation between these factors, with a higher EWL% in patients with greater RGV [5].

Singh et al. in their study included 100 bariatric patients undergoing LSG. They were divided into three groups according to the volume of the resected stomach and EWL% recorded at each follow-up. They concluded that EWL% was not significantly different among the groups and then not influenced by the RGV [4].

Our study is in line with these last results. The lack of correlation could imply that the volume of the sleeve is more important than the amount of stomach removed. Moreover, for the weight regain, probably, the dilatation of the gastric tube in the midlong-term is the primary factor, rather than the RGV following LSG. For this reason, we think that patients need to be under strict nutritional control in the long-term, not only during the first period after LSG.

We acknowledge some limitations of our study. It is a retrospective analysis and the number of patients is not very large. Further studies, maybe applying the same mathematical method, and longer follow-up, will be useful in the future in order to clarify the potential role of the RGV in the weight loss after LSG.

## 5. Conclusion

Measurement of the resected gastric volume needs a standard and validated procedure. Our mathematical method could represent a reproducible tool to investigate this topic.

From the results of this study, there is no correlation between the volume of stomach removed and the weight loss at 1 year after LSG. Further researches in the future could corroborate and clarify this relationship.

## Figures and Tables

**Table 1:** Preoperative data.

		*N*	Mean	Std. deviation	Std. error	95% confidence interval for mean		Minimum	Maximum
						Lower bound	Upper bound		
Height (cm)	F	44	163.65	6.36	0.959	161.71	165.58	150	176
	M	26	179.38	8.84	1.735	175.81	182.96	164	195
	Total	70	169.49	10.59	1.266	166.97	172.02	150	195

Weight (Kg)	F	44	119.20	15.33	2.312	114.54	123.87	93	160
	M	26	155.54	26.94	5.284	144.66	166.42	110	205
	Total	70	132.70	26.87	3.212	126.29	139.11	93	205

BMI	F	44	44.34	5.2	0.77965	42.77	45.91	37.00	57.80
	M	26	47.18	7.9	1.54849	43.99	50.37	38.00	67.47
	Total	70	45,39	6.4	0.76693	43.86	46.92	37.00	67.47

**Table 2:** Postoperative data after 1-year from LSG.

		*N*	Mean	Std. deviation	Std. error	95% confidence interval for mean		Minimum	Maximum
						Lower bound	Upper bound		
Ideal weight (Kg)	F	44	56.80	3.16	0.476	55.84	57.76	50	63
	M	26	72.05	6.64	1.302	69.37	74.73	61	84
	Total	70	62.46	8.79	1.051	60.37	64.56	50	84

BMI 1 year	F	44	29.836	5.06	0.7634	28.29	31.37	19.6	42,0
	M	26	30.608	5.83	1.1452	28.25	32.96	20.7	43.5
	Total	70	30.123	5.33	0.6378	28.85	31.39	19.6	43.5

Weight 1 year	F	44	80.07	13.75	2.074	75.89	84.25	57	110
	M	26	98.92	23.64	4.636	89.38	108.47	63	157
	Total	70	87.07	20.11	2.404	82.28	91.87	57	157

EWL% 1 year	F	44	62.82	19.75	2.97	56.82	68.82	23.33	104.36
	M	26	69.04	20.87	4.09	60.61	77.47	20.47	108.71
	Total	70	65.13	20.25	2.42	60.30	69.96	20.47	108.71

**Table 3:** Correlation between RGV, EWL%, and BMI at 1 year after surgery.

		EWL% 1 aa	Sex	Volume resected (mL)	BMI 1 aa
EWL% (1 year)	Pearson correlation	1	0.149	0.167	-0.872(^∗∗^)
	Sig. (2-tailed)		0.217	0.168	0.000
	*N*	70	70	70	70

Sex	Pearson correlation	0.149	1	0.075	0.070
	Sig. (2-tailed)	0.217		0.536	0.563
	*N*	70	70	70	70
	*N*	70	70	70	70

Resected gastric volume (mL)	Pearson correlation	0.167	0.075	1	-0.172
	Sig. (2-tailed)	0.16S	0.536		0.156
	*N*	70	70	70	70

BMI (1 year)	Pearson correlation	-0.872(^∗∗^)	0.070	-0.172	1
	Sig. (2-tailed)	0.000	0.563	0.156	
	*N*	70	70	70	70

^∗∗^Correlation is significant at the 0.01 level (2-tailed). ^∗^Correlation is significant at the 0.05 level (2-tailed).

## Data Availability

The datasets generated during and/or analyzed during the current study are available from the corresponding author on reasonable request.
